# Chimeric single-chain variable fragment-human immunoglobulin G crystallizable fragment antibody against GD2 for neuroblastoma targeted immunotherapy

**DOI:** 10.37349/etat.2023.00188

**Published:** 2023-12-06

**Authors:** Witida Laopajon, Nuchjira Takheaw, Kamonporn Kotemul, Supansa Pata, Suradej Hongeng, Watchara Kasinrerk

**Affiliations:** Istituto Nazionale Tumori-IRCCS-Fondazione G. Pascale, Italy; ^1^Division of Clinical Immunology, Department of Medical Technology, Faculty of Associated Medical Sciences, Chiang Mai University, Chiang Mai 50200, Thailand; ^2^Biomedical Technology Research Center, National Center for Genetic Engineering and Biotechnology, National Science and Technology Development Agency at the Faculty of Associated Medical Sciences, Chiang Mai University, Chiang Mai 50200, Thailand; ^3^Department of Pediatrics, Faculty of Medicine Ramathibodi Hospital, Mahidol University, Bangkok 10400, Thailand

**Keywords:** Neuroblastoma, disialoganglioside, single-chain variable fragment-fragment crystallization region fusion antibody, targeted immunotherapy

## Abstract

**Aim:**

The present study aims to generate chimeric mouse single-chain variable fragment (scFv) and immunoglobulin G1 (IgG1) crystallizable fragment (Fc) antibody against disialoganglioside (GD2) for the treatment of neuroblastoma (NB). The generated scFv-IgG Fc antibody, lacking first constant domain of heavy chain (CH1), is of a smaller size than the natural antibody and has anti-tumor activity.

**Methods:**

Vector for scFv-IgG Fc antibody was constructed and scFv-IgG Fc antibody was expressed in human embryonic kidney 293T (HEK293T) cell line. Purification of scFv-IgG Fc antibody from the culture supernatant of transfected HEK293T cells was performed by Protein G affinity chromatography. The structure and binding activity of scFv-IgG Fc antibody were verified by sodium dodecyl sulfate-polyacrylamide gel electrophoresis (SDS-PAGE), western blotting (WB), and immunofluorescence techniques. Anti-tumor activities by antibody-dependent cellular cytotoxicity (ADCC) and antibody-dependent cellular phagocytosis (ADCP) were determined.

**Results:**

Using plasmid fusion-human IgG1-Fc2 tag vector (pFUSE-hIgG1-Fc2), a plasmid vector encoding chimeric mouse scFv and hIgG1 Fc antibody against GD2 was successfully constructed. This vector was transfected into human HEK293T cells to produce scFv-IgG Fc antibody. The transfected HEK293T cells could produce chimeric scFv-IgG Fc antibody against GD2, which lacks the IgG heavy chain CH1 domain but carries CH2 and CH3 domains. The chimeric antibodies could be purified from the culture supernatant of the transfected HEK293T culture in the presence of zeocin drug. The produced GD2 scFv-IgG Fc antibodies, which are smaller in size than the intact antibody, could trigger the killing of GD2 expressed NB cell line SH-SY5Y by ADCC and ADCP mechanisms.

**Conclusions:**

The results indicate that chimeric scFv-hIgG Fc antibody, lacking heavy chain CH1 domain, could mediate antibody induced anti-tumor activities. The small size of this type of chimeric antibody may be employed as anti-GD2 antibody for NB therapy.

## Introduction

Neuroblastoma (NB) is the most common extracranial solid tumor in children and accounts for up to 15% of deaths in children’s cancers [1, 2]. Neural crest cells were proposed as the origin of NB [3]. The most common primary site for NB is the adrenal gland. NB occurs along the sympathetic nervous system leading to the formation of cancerous tissues, usually prevailing during the development of neuroblasts in the embryonic neural crest [3]. NB patients are subdivided into low-, intermediate- and high-risk groups [4]. Patients with low- and intermediate-risk NB have favorable prognoses and an excellent five-year survival rate of more than 90%. In contrast, the prognosis of treatment remains unfavorable in the case of high-risk NB (HR-NB) [5]. HR-NB patients have poor overall survival (OS) despite intensive therapy. Unexpectedly, the proportion of HR-NB cases in Thailand was determined as 85% higher than cases in Western countries [6]. Standard treatment for HR-NB patients includes surgery, radiation, and/or myeloablative chemotherapy with autologous stem cell transplantation. Even if this multimodal therapy were used, the five-year survival rate remains under 50% with strong side effects [3, 5]. In recent years, immunotherapy has become a promising approach for the treatment of several cancers, including HR-NB [7].

Disialoganglioside (GD2) is a glycolipid GD2 antigen that is expressed on tumors of neuroectodermal origin, including NB [8‒10]. GD2 is highly expressed on NB cell membranes regardless of stage and showed heterogenous expression pattern among individual patients [11, 12]. In addition, the GD2 expression levels were not reduced after antibody targeting [13]. In contrast, in normal tissues, GD2 expression is restricted to peripheral neurons, the central nervous system, and skin melanocytes [14]. Thus, it is well suitable for targeted antitumor therapy with less cytotoxicity to normal tissues. These properties, therefore, make it an attractive target for NB immunotherapy. Since the discovery of anti-GD2 monoclonal antibodies (mAbs) in 1985 by Cheung and colleagues [9], several formats of GD2-specific mAbs have been developed for the treatment of NB. The GD2-specific chimeric antibody combined with interleukin-2 (IL-2) and granulocyte-macrophage colony stimulating factor (GM-CSF) was approved by the European Medicines Agency (EMA) and US Food and Drug Administration (FDA) in 2015 for the treatment of HR-NB [15, 16]. Dinutuximab (trade name Unituxin) represents human-mouse chimeric mAb ch14.18. The ch14.18 was constructed by combining the variable region of murine anti-GD2 mAb and the constant region of human immunoglobulin G1 (IgG1; hIgG1). Ch14.18 preserves the binding properties of the antibody to the GD2 ganglioside [17, 18]. HIgG crystallizable fragment (Fc) part is useful for mediating immune effectors, including complement-dependent cytotoxicity (CDC), antibody-dependent cellular phagocytosis (ADCP), and antibody-dependent cellular cytotoxicity (ADCC) [19, 20]. Dinutuximab significantly increases the survival of HR-NB patients [16]. However, the maximal efficacy of this therapy is yet to be achieved. Dinutuximab is an intact antibody, it is probably better to produce a smaller antibody size with the same antigen binding activity. The smaller size of antibody might penetrate tumor sites more efficiently [21]. This may enhance the efficacy of desirable antitumor effects. Thus, this study aimed to develop novel GD2-specific antibodies for NB therapy.

In this study, the chimeric mouse GD2 single-chain variable fragment (scFv)-hIgG1 Fc antibody was generated. This chimeric antibody has no antibody first constant domain of heavy chain (CH1). The remaining Fc CH2 and CH3 domains of hIgG in the generated chimeric antibody still have efficacy in mediating immune effectors via ADCC and ADCP. The generated chimeric antibody has a smaller size than commercial human-mouse chimeric mAb ch14.18. This would diffuse into tumor tissue more efficiently.

## Materials and methods

### Construction of scFv-IgG Fc plasmid vectors

Nucleotide sequence of mouse scFv against GD2 clone 14G2a (a kind gift from Prof. Dr. Suradej Hongeng, Department of Pediatrics, Faculty of Medicine, Ramathibodi Hospital, Mahidol University, Bangkok, Thailand) was designed to be inserted into mammalian expression plasmid fusion-hIgG1-Fc2 tag vector (pFUSE-hIgG1-Fc2) using SnapGene software (version 1.1.3, GSL Biotech LLC, San Diego, CA, USA). The gene encoding scFv against GD2 was synthesized and amplified by polymerase chain reaction (PCR) using forward primer with EcoRI site; 5’-GAGGAGGAATTCggatattttgctgacccaaact-3’ and reverse primer with NcoI site; 5’-GAGGAGCCATGGctgaggagacggtgact-3’. The PCR products of gene encoding scFv against GD2 were cloned into vector pFUSE-hIgG1-Fc2 (InvivoGen, Toulouse, France). The genes were inserted between IL-2 signal sequence (IL2ss) and the hinge region of the hIgG1 Fc part (CH2-CH3 domain). The constructed vector was named pFuse-scFv-GD2-hIgG1-Fc2 vector. The constructed vectors were verified by DNA sequencing. The synthesis of nucleotide sequence of mouse scFv against GD2, amplification, cloning, and DNA sequencing processes were carried out by Bio Basic Inc. (Singapore).

To increase the vector yield for expression of GD2 specific scFv-IgG Fc antibody, the pFUSE-scFv-GD2-hIgG1-Fc2 vector was transformed into competent *Escherichia coli* (*E. coli*) DH5-α. *E. coli* carrying vectors were selected by spreading on Luria-Bertani (LB) agar containing 25 µg/mL of zeocin (cat# ant-zn-1p, InvivoGen). The single colonies were picked up and the *GD2* gene was verified by PCR. A bacterial clone containing constructed plasmid vectors was cultured in LB broth containing 25 µg/mL of zeocin at large scale. The plasmid vectors were extracted using plasmid midi preparation kit (cat# K0481, Thermo Fisher Scientific Baltics UAB, Vilnius, Lithuania).

### Production of scFv-IgG Fc antibody against GD2 in mammalian expression system

The generated pFuse-scFv-GD2-hIgG1-Fc2 vectors were transfected into human embryonic kidney 293T (HEK293T) cell line. In brief, cells (2 × 10^5^ cells) were plated into 6-well plate (cat# 3516, Corning Inc., NY, USA) and incubated at 37^°^C, 5% CO_2_ for 2 days. The plasmid vector (500 ng) was incubated with 3 µL of Lipofectamine® 2000 reagent (cat# 11668-027, Invitrogen, Carlsbad, CA, USA) at room temperature for 20 min and added into HEK293T cells. After transfection, the cells were incubated at 37^°^C, 5% CO_2_ for three days. The transfected cells were harvested and intracellular immunofluorescence was stained to determining protein expression.

For staining intracellular protein expression, cells were fixed with 4% paraformaldehyde (cat# A11313, Alfa Aesar, Ward Hill, MA, USA) and permeabilized with 0.1% saponin (cat# 97061-114, VWR Life Science, Radnor, PA, USA). Later, cells were blocked at the Fc receptor using 10% fetal bovine serum (FBS, cat# A5256701, Gibco, GrandIsland, NY, USA) and stained with 50 µL of Alexa Fluor 488 conjugated goat anti-hIgG antibody (1:500, cat# 109-545-098, Jackson ImmunoResearch Laboratories, West Grove, PA, USA) or Alexa Fluor 488-conjugated goat anti-mouse IgG antibody (1:250, cat# A11029 Life technology, Eugene, OR, USA) as a control. The expression of scFv-Fc antibody was determined by flow cytometry (Accuri^TM^ C6 flow cytometer, BD Biosciences, San Jose, CA, USA).

For selection of stable expression of HEK293T cells, the successfully transfected HEK293T cells were plated at 100 cells in 10% FBS-Dulbecco’s Modified Eagle Medium (DMEM, cat# 12800-017, Gibco) containing 100 µg/mL of zeocin drug in a 96-well plate (cat# 3599, Corning Inc). Cells that received vector harboring zeocin drug resistant gene were grown. Cells were screened for the expression of scFv-IgG Fc antibody against GD2 by intracellular immunofluorescence staining as described above.

### Large-scale production and purification of scFv-IgG Fc antibody

The HEK293T clone expressing scFv-IgG Fc antibody against GD2 was seeded in T 75 cm^2^ flask (cat# 156499, Thermo Scientific, Rockford, IL, USA) and grown in 10% FBS-DMEM containing 100 µg/mL zeocin. After cells reached 70% confluence, culture media was removed and gently washed with DMEM for 3 times. The media were then replaced with Chinese Hamster Ovary-Suspension-Serum Free Media II (CHO-S-SFM II, cat# 12052-098, Gibco). Cells were cultured in serum-free media containing 100 µg/mL zeocin at 37^°^C in 5% CO_2_ for 72 h. Culture supernatant was harvested. Two hundred milliliters of culture supernatant was subjected to purification of the scFv-IgG Fc antibody by affinity chromatography using HiTrap Protein G column (cat# 17-0404-03, GE Healthcare, Uppsala, Sweden). The concentration of purified antibody was measured by bicinchoninic acid assay (BCA) protein assay kit (cat# 23227, Thermo scientific). The purity and structure of purified antibodies were validated by SDS-PAGE and western blotting (WB) using horseradish peroxidase (HRP)-conjugated rabbit anti-human Ig antibodies (cat# P0212, Dako, Glostrup, Denmark). The binding activity of purified antibodies was also determined by immunofluorescence staining using GD2 expressing cells.

### Validation of the binding activity of purified scFv-IgG Fc antibody against GD2

Human NB cell line, SH-SY5Y, which expressed GD2 on cell surface, was stained with purified scFv-Fc antibodies. The bound scFv-Fc antibodies were detected by Alexa Fluor 488-conjugated anti-hIgG antibody. The binding activity of antibodies was analyzed by flow cytometry.

### ADCC assay

Peripheral blood was collected from healthy donors using sodium heparin as an anti-coagulant or buffy coat of healthy individuals obtained from The Reginal Blood Center X, Thai Red Cross Society, Chiang Mai, Thailand. Peripheral blood mononuclear cells (PBMCs) were isolated by Ficoll-Hypaque density gradient centrifugation.

For ADCC assay, SH-SY5Y was labeled with carboxyfluorescein diacetate succinimidyl ester (CFSE, cat# 21888, Sigma-Aldrich, Saint Louis, MO, USA) and used as a target cell. CFSE labeled SH-SY5Y was incubated with or without purified GD2-scFv-IgG Fc antibodies and co-cultured with PBMCs (effector cells) at various effector cell: target cell (E:T) ratios or without effector cells. After being incubated for 4 h at 37^°^C in a 5% CO_2_ incubator, cells were harvested, washed twice with 200 µL of phosphate buffered saline (PBS, cat# 524650, EMD Millipore, Burlington, MA, USA), and then stained with 200 µL of propidium iodide (PI, cat# P4170, Sigma-Aldrich) solution at final concentration of 2 µg/mL. The percentage of dead target cells (CFSE^+^PI^+^) was determined using an Accuri C6 flow cytometer (BD Biosciences).

### ADCP assay

Human monocyte-enriched PBMCs were prepared from PBMCs by Percoll gradient centrifugation. Briefly, PBMCs were diluted with PBS at a concentration of 1 × 10^7^ cells/mL. Then, diluted PBMCs were overlaid on Percoll solution (cat# 17-0891-02, GE Healthcare) and centrifugated at 25^°^C, 895 *g*, for 40 min. After centrifugation, the monocyte-enriched PBMCs were collected and washed. Thereafter, the monocyte-enriched PBMCs were determined for a percentage of monocyte using flow cytometry. The obtained monocyte-enriched PBMCs were plated in 24-well plates (cat# 3524, Corning Inc) at the number of monocytes of 5 × 10^5^ cells per well and incubated in a 5% CO_2_ incubator for 2 h. Then, non-adherent cells were removed by washing with PBS and macrophage colony-stimulating factor (M-CSF, cat# 11343113 Immuno Tools, Friesoythe, Germany), 50 ng/mL, was added and incubated for six days to induce macrophage differentiation. Upon culturing, 50 ng/mL M-CSF was added every two days.

For ADCP assay, SH-SY5Y cells were labeled with CFSE (molecular probes). The CFSE labeled SH-SY5Y cells were incubated with or without purified GD2-scFv-IgG Fc antibodies. Monocyte-derived macrophages (effector cells) were added into CFSE labeled SH-SY5Y cells (target cells) and cultured in a 96-well flat plate at various E:T ratios. The co-cultured cells were incubated in a humidified atmosphere of 5% CO_2_ at 37^°^C for 30 min. After cultivation, cells were harvested and stained with PerCP-conjugated anti-CD14 mAb (cat# 325632, BioLegend, San Diego, CA, USA). Then, the cells were analyzed by flow cytometry for assessing the percentage of phagocytosed cells (CFSE^+^CD14^+^ cells).

### Statistical analysis

All statistical analyses were performed using GraphPad Prism version 9.2.0 (GraphPad Software, CA, USA). One-way analysis of variance (ANOVA) was used as indicated in the figure legends. Statistical significance was accepted at a *P* value < 0.05.

## Results

### Construction of scFv-IgG Fc plasmid vectors

**Figure 1:**
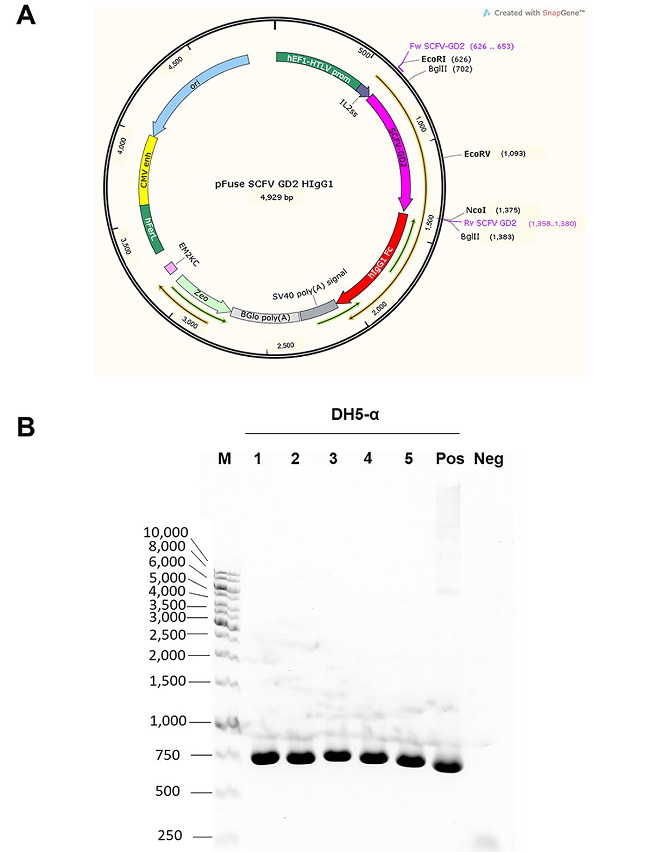
Graphical map of the constructed plasmid vector and PCR analysis of *GD2 scFv* gene. (A) Graphical map of pFUSE-hIgG1-Fc2 vector created by SnapGene software is shown. Nucleotide sequence of mouse scFv against GD2 was cloned into the vector at EcoRI and NcoI sites before the hinge region of the hIgG1 Fc part. The thin orange arrow indicates an open reading frame, which starts at the codon inside IL2ss and stops at the end of the Fc portion. Zeocin drug resistance gene (*Zeo*) was used for selection; (B) after cloning step, the constructed vector was transformed into DH5-α *E. coli* and selected by zeocin drug. Five bacterial colonies (as indicated) were picked up and grown. PCR was performed to detect the inserted *GD2 scFv* gene inside the constructed plasmid vectors. Bacteria clone number 1 was chosen for plasmid extraction. Standard DNA markers (M) in base pairs are shown on the left. Positive and negative controls are also demonstrated. Neg: negative; Pos: positive

In this study, we aimed to generate a chimeric antibody, which consists of mouse scFv against GD2 and hIgG1 Fc domain. This chimeric antibody was designed to lack heavy chain CH1 domain; thus, having a smaller size than the natural intact antibody. In order to obtain the chimeric antibody, the vector containing scFv-GD2 fused with hIgG1 Fc part was constructed. The graphical map of the constructed plasmid vector is shown in Figure 1A. The inserted genes were verified by DNA sequencing and showed 100% identity match to the original sequence. After transformation and dug selection, five single bacterial colonies were picked up and verified gene encoding scFv GD2. The PCR products at 741 base pairs (bp), as expected, were observed in all selected bacterial clones (Figure 1B). The plasmid vectors were used to express further.

### Production of scFv-IgG Fc antibody against GD2 in mammalian cells

**Figure 2:**
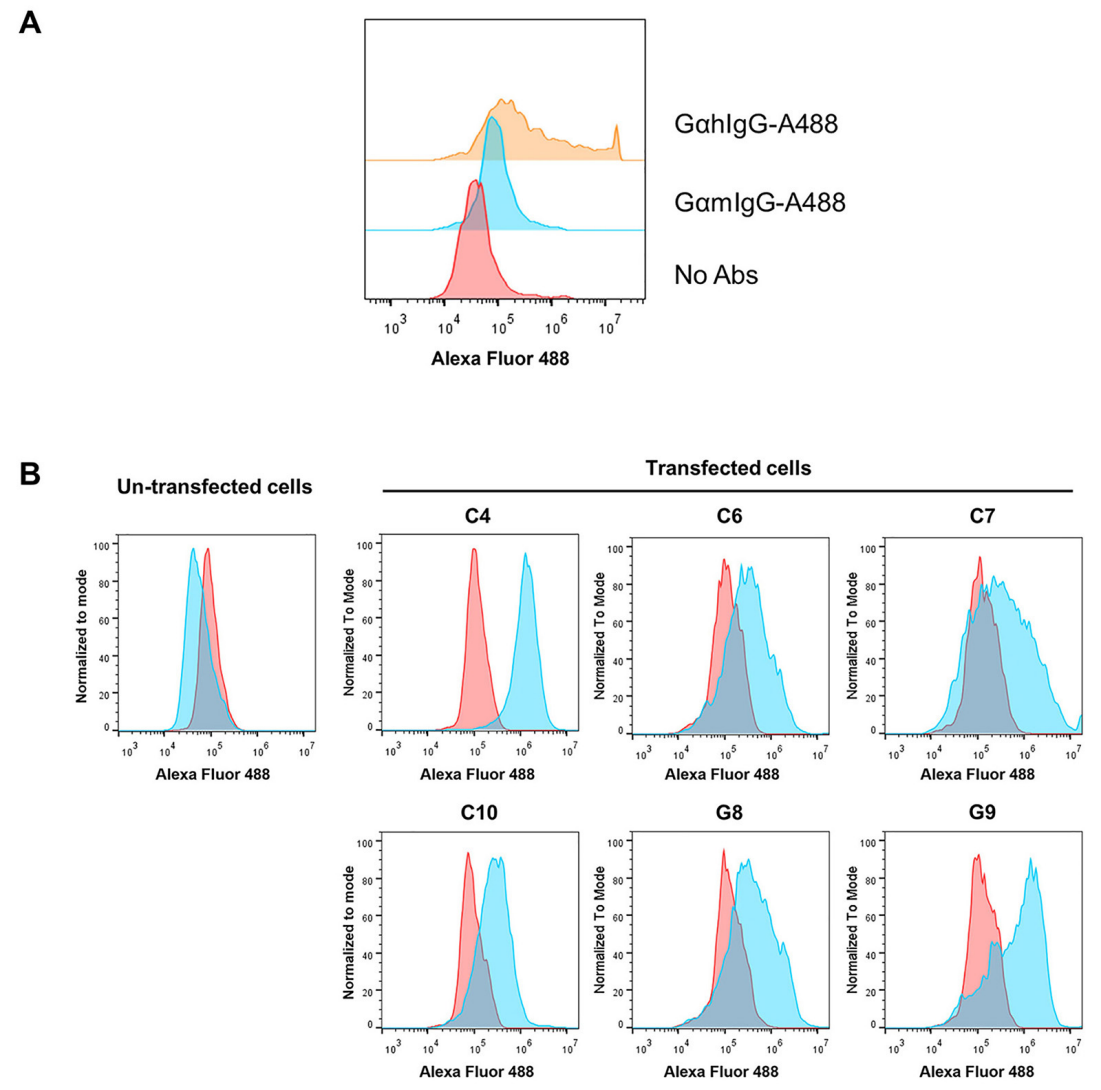
Determination of scFv-IgG Fc expression in transfected HEK293T cells. (A) Human HEK293T cell line was transfected with pFUSE-scFv-GD2-hIgG1-Fc2 vector and intracellular stained with Alexa Fluor 488-conjugated goat anti-hIgG antibody (GαhIgG-A488), Alexa Fluor 488-conjugated goat anti-mouse IgG antibody (GαmIgG-A488), or without antibodies (no Abs); (B) transfected HEK293T cells were cultured in the presence of zeocin. The transfected cells and un-transfected cells were intracellularly stained with Alexa Fluor 488-conjugated goat anti-hIgG antibody (Blue color) or Alexa Fluor 488-conjugated goat anti-mouse IgG antibody (red color). Overlay histograms are shown

In order to establish stable-expressing mammalian cell line mouse scFv-hIgG1 Fc antibody against GD2, human HEK293T cell line was used as a host cell. Three days after transfection, transfected cells were intracellular immunofluorescence stained. The flow cytometric analysis showed that approximately 50% of transfected cells were positive with anti-hIgG antibody, but negative in anti-mouse IgG antibody control (Figure 2A).

Then, six transfected HEK293T containing wells were harvested to determine the expression of the scFv-IgG Fc antibody. All selected cells showed positive reactivity with Alexa Fluor 488-conjugated anti-hIgG antibody, but at different expression levels (Figure 2B). Reactivity with Alexa Fluor 488-conjugated anti-mouse IgG antibody was not detected. Un-transfected cells were also included in the experiment and showed negative reactivity with all Alexa Fluor 488-conjugated antibodies. These results indicated that stable human HEK293T cells expressing scFv-hIgG Fc antibodies were established. The transfected HEK293T well named C4, which had the highest expression level (100% positive), was selected to produce scFv-IgG Fc antibodies.

### Purification and large-scale production of scFv-IgG Fc against GD2

**Figure 3:**
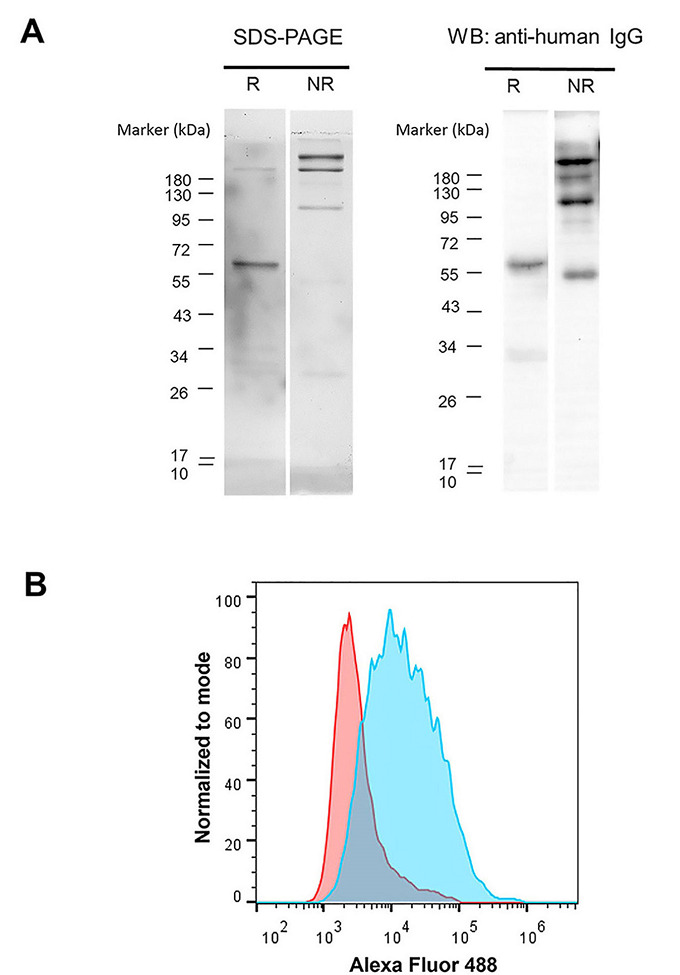
Validation of the purity, structure, and activity of purified GD2 scFv-hIgG Fc antibody. (A) Purified GD2 scFv-IgG Fc antibody was resolved in 10% SDS-PAGE under reducing (R) conditions and non-reducing (NR) conditions. Protein bands were stained with Coomassie blue. WB analysis was performed using HRP conjugated anti-hIgG antibody. The molecular weight (kDa) of protein markers is shown on the left; (B) human NB cell line (SH-SY5Y cell) was stained with purified GD2 scFv-IgG Fc antibody (blue color) or irrelevant IgG Fc fusion control protein (red color) followed by Alexa Fluor 488-conjugated goat anti-hIgG antibody and analyzed by flow cytometry. The overlay histogram is shown

The scFv-IgG Fc in the serum-free culture supernatant was purified by Protein G column. The purity and structure of purified scFv-IgG Fc were investigated. As shown in Figure 3A, by SDS-PAGE, the size of scFv-IgG Fc antibody in reducing conditions was monomeric at approximately 60 kilodalton (kDa). In non-reducing conditions, the majority band at 120 kDa, which was expected to be dimeric form, was observed. Unpredictable bands at 180 kDa and higher were also found. These bands were likely trimeric and tetrameric scFv-IgG Fc antibodies. The bands in SDS-PAGE were closely related to those observed in WB using anti-hIgG antibody.

Furthermore, the binding activity of the purified proteins was determined by staining with GD2 expressing SH-SY5Y cells [21]. The purified GD2 scFv-IgG Fc antibody showed positive reactivity to SH-SY5Y cells (Figure 3B). These results indicated that the scFv-hIgG Fc fusion antibody against GD2 could be produced and secreted by HEK293T cells. The purified scFv-IgG Fc antibody could be prepared from the HEK293T culture supernatant.

### Anti-tumor activities of scFv-IgG Fc antibody against GD2

**Figure 4:**
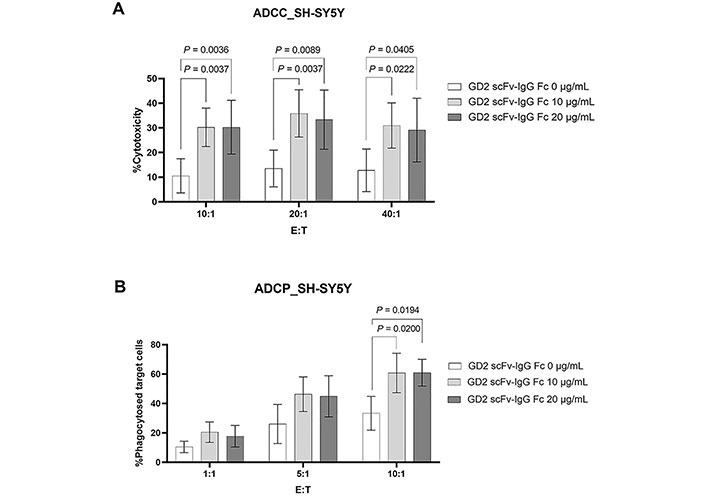
ADCC and ADCP activities mediated by the generated scFv-IgG Fc antibody against GD2. (A) CFSE labeled SH-SY5Y cells were incubated with or without the generated scFv-IgG Fc antibody at 10 µg/mL and 20 µg/mL and PBMCs (*n* = 6) were added at the indicated E:T ratio. The dead target cells (CFSE^+^PI^+^) were analyzed by flow cytometry. Percent cytotoxicity was calculated as [dead target cells (%) − spontaneous death (%)]/(100 − spontaneous death); (B) for ADCP, macrophages (*n* = 4) were used instead of PBMCs and the percentage of phagocytosed target cells (CFSE^+^ CD14^+^ cells) is shown. Data normality was assessed using the Shapiro–Wilk test. All data sets were normally distributed except ADCP at E:T ratio of 10:1 in condition with GD2 scFv-IgG Fc 20 μg/mL. The values are shown as mean ± SD. One**-**way ANOVA followed by Tukey**’**s multiple comparison test was used for comparison

The GD2 expression cell line SH-SY5Y was used as target cell for determining antitumor activities. In ADCC assay, scFv-IgG Fc antibody at 10 µg/mL and 20 µg/mL significantly killed GD2 expressing SH-SY5Y cells [22]. This effect was observed at all tested E:T ratios (10:1, 20:1, 40:1) and compared with zero scFv-IgG Fc antibodies (0 µg/mL) as demonstrated in Figure 4A.

In addition, the activity of the generated scFv-IgG Fc antibody in ADCP was determined. As shown in Figure 4B, macrophages could phagocytose SH-SY5Y upon being sensitized with the generated scFv-IgG Fc antibody at 10 µg/mL and 20 µg/mL. This was statistically significant at an E:T ratio of 10:1. At lower E:T ratios (1:1 and 5:1), an increasing trend of ADCP could be observed but it was not statistically significant.

Taken together, the results demonstrated that the generated scFv-IgG Fc antibody against GD2 has anti-tumor activities in the induction of both ADCC and ADCP.

## Discussion

NB is a malignancy of the sympathetic nervous system that almost exclusively occurs in early childhood. The formation of cancerous tissues usually prevails during the development of an immature nerve cell called a neuroblast in the embryonic neural crest, which finally differentiates into the cell lineages of sympathetic ganglia and adrenal gland [23]. Standard treatment for NB patients, particularly HR-NB, is unsatisfied.

GD2 is a tumor-associated antigen that was demonstrated to express highly in NB cells. However, GD2 is restricted in normal tissue [14]. Thus, GD2 is a promising target for immunotherapy in NB treatment. Currently, a chimeric mouse-human GD2-specific antibody was developed for use in NB targeted immunotherapy [15, 16]. This antibody shows high efficacy in NB therapy, but less cytotoxicity to normal tissues. To date, several formats of the GD2-specific monoclonal antibody have been developed for the treatment of NB. However, the maximal efficacy of therapy is yet to be achieved. The appropriate glycosylation of IgG1 Fc part in the human expression system is still needed for activating immune effectors. Moreover, it has been demonstrated that a smaller antibody size is very useful for penetrating tumor sites with the same antigen binding [21].

In this study, we generated the chimeric mouse GD2 scFv-hIgG1 Fc antibody. By the vector used [24], the generated chimeric antibody lacked the CH1 domain of the heavy chain. This made the generated antibody a smaller size than the native antibody. The antibody was produced in human HEK293T cells, designed to have the correct hIgG Fc part. The chimeric antibodies could be produced in human HEK293T cells and purified from the constructed HEK293T cell culture supernatants.

By SDS-PAGE, WB, and immunofluorescence staining, the purified scFv-IgG Fc antibody was proven to have a corrected structure, was able to react to GD2 expressing cells, and contained IgG Fc. According to the vector used, the chimeric mouse GD2 scFv-hIgG1 Fc antibody has no heavy chain CH1 part. However, the remaining Fc CH2 and CH3 domains of hIgG would mediate immune effectors.

Currently, the ADCC and ADCP mechanisms have been demonstrated to be important immune mechanisms for cancer therapy [25, 26]. This study examined whether the generated chimeric mouse GD2 scFv-hIgG1 Fc antibody could mediate ADCC and ADCP. It was found that the generated scFv-IgG Fc antibody against GD2 exerted potent ADCC and ADCP activities in GD2-expressing NB cells. The results indicated that a chimeric antibody, even lacking heavy chain CH1 domain, could mediate antibody induced anti-tumor activities. The small size of this type of chimeric scFv-IgG Fc antibody might be useful as alternative anti-GD2 antibody for NB therapy.

## Abbreviations

ADCC: antibody-dependent cellular cytotoxicity
ADCP: antibody-dependent cellular phagocytosis
CFSE: carboxyfluorescein diacetate succinimidyl ester
CH1: first constant domain of heavy chain
Fc: crystallizable fragment
GD2: disialoganglioside
HEK293T: human embryonic kidney 293T
hIgG1: human immunoglobulin G1
HR-NB: high-risk neuroblastoma
IgG1: immunoglobulin G1
kDa: kilodalton
mAb: monoclonal antibody
NB: neuroblastoma
PBMCs: peripheral blood mononuclear cells
PCR: polymerase chain reaction
pFUSE-hIgG1-Fc2: plasmid fusion human immunoglobulin G1 crystallizable fragment 2 tag vector
PI: propidium iodide
scFv: single-chain variable fragment
WB: western blotting

## Declarations

### Availability of data and materials

The datasets are in the manuscript, and requests for details should be directed to the corresponding author (watchara.k@cmu.ac.th).

### Copyright

© The Author(s) 2023.
